# On the Quantumness of Multiparameter Estimation Problems for Qubit Systems

**DOI:** 10.3390/e22111197

**Published:** 2020-10-23

**Authors:** Sholeh Razavian, Matteo G. A. Paris, Marco G. Genoni

**Affiliations:** 1 Faculty of Physics, Azarbaijan Shahid Madani University, Tabriz 5375171379, Iran; sholeh.razavian@gmail.com; 2 Quantum Technology Lab, Dipartimento di Fisica “Aldo Pontremoli”, Università degli Studi di Milano, I-20133 Milano, Italy; 3 INFN, Sezione di Milano, I-20133 Milano, Italy; matteo.paris@fisica.unimi.it

**Keywords:** quantum sensing, quantum metrology, quantum probes, multiparameter estimation

## Abstract

The estimation of more than one parameter in quantum mechanics is a fundamental problem with relevant practical applications. In fact, the ultimate limits in the achievable estimation precision are ultimately linked with the non-commutativity of different observables, a peculiar property of quantum mechanics. We here consider several estimation problems for qubit systems and evaluate the corresponding *quantumness* R, a measure that has been recently introduced in order to quantify how incompatible the parameters to be estimated are. In particular, R is an upper bound for the renormalized difference between the (asymptotically achievable) Holevo bound and the SLD Cramér-Rao bound (i.e., the matrix generalization of the single-parameter quantum Cramér-Rao bound). For all the estimation problems considered, we evaluate the quantumness R and, in order to better understand its usefulness in characterizing a multiparameter quantum statistical model, we compare it with the renormalized difference between the Holevo and the SLD-bound. Our results give evidence that R is a useful quantity to characterize multiparameter estimation problems, as for several quantum statistical model, it is equal to the difference between the bounds and, in general, their behavior qualitatively coincide. On the other hand, we also find evidence that, for certain quantum statistical models, the bound is not in tight, and thus R may overestimate the degree of quantum incompatibility between parameters.

## 1. Introduction

Quantum sensing is the art of exploiting quantum features as coherence (or decoherence) to improve the sensitivity of measuring devices [1,2,3,4,5,6,7,8,9,10,11]. The field originates from fundamental research in the detection of gravitational waves, and is now a successful quantum technology. When the problem at hand involves a single parameter, there is a clear avenue to define optimality: build a suitable quantum statistical model, evaluate the symmetric logarithmic derivative (SLD) operator and then the corresponding quantum Cramér-Rao SLD-bound. The SLD-bound indeed sets the ultimate precision achievable by any estimation strategy, and links it to the quantum Fisher Information (QFI), which quantifies the amount of information extractable via quantum measurements on the parameter. Quantum probes and detectors are then designed to maximize and possibly attain the QFI for given resources, possibly outperforming any conceivable setup using only classical states and detectors.

As a matter of fact, there are several problems of interest that are inherently involving more than one parameter [12,13,14,15], e.g. estimation of unitary operations and of multiple phases [16,17,18,19,20,21,22,23,24,25,26], estimation of phase and noise [27,28,29,30], and superresolution of incoherent sources [31,32,33]. However, despite these important applications, multiparameter quantum estimation received less attention and some relevant (fundamental and practical) issues have not yet fully resolved. In particular, the non-commutativity of the quantum observables needed to jointly estimate a vector of parameters poses non-trivial limitations. As a result, despite its classical counterpart being asymptotically achievable, non-commutativity [34,35], makes the multiparameter SLD-bound [36,37] generally not achievable. Indeed, the multiparameter estimation bounds are given by matrix inequalities which are not tight. In order to access the fundamental precision limit in the multiparameter quantum estimation problem, Holevo proposed a scalar bound [38,39]. The Holevo bound is regarded as the most fundamental scalar lower bound, as it is attainable by allowing collective measurements on an asymptotically large number of copies of the quantum state defining the quantum statistical model [40,41]. Besides the practical applications, the difference between the Holevo- and the SLD-bound also allows for assessing the degree of *incompatibility* of the parameters to be estimated. Nevertheless, the expression of the Holevo bound is not crystal clear in terms of the model under consideration, since it is written as an optimization over a set of matrices.

We take the simplest quantum systems, a qubit, and consider several multiparameter estimation problems involving unitary and noisy channels. For those models, we evaluate its *quantumness* R that has been recently introduced in [42]. This quantity is supposed to estimate the amount of the incompatibility of the parameters defining the quantum statistical model and can be easily evaluated via the Uhlmann curvature matrix and the SLD-QFI matrix. Then, we also evaluate the Holevo bound that, for the case of two-parameter qubit models, can be evaluated analytically via the SLD and the right logarithmic derivative (RLD) operators only [43]. In particular, we compare R with the renormalized difference $\Delta C_{\max}$ between the Holevo and the SLD-bound, maximized over all the possible weight matrices, to assess its performance as an upper bound. Our results show that the two quantities always share the same qualitative behavior, but also give evidence that the bound is not always tight, i.e., R is a useful quantity to characterize multiparameter estimation problems, but it may overestimate the incompatibility between parameters.

The paper is structured as follows. In Section 2, we briefly review the theory behind multi-parameter quantum metrology, we introduce the measure for *quantumness* R and discuss its main properties. In Section 3, we evaluate the quantumness parameter and the renormalized difference between Holevo- and SLD-bound for several multiparameter quantum statistical models for single qubits, discussing their relationship in more detail. Section 4 concludes the manuscript with a final discussion.

## 2. Multi-Parameter Quantum Metrology and a Measure of *Quantumness* for Quantum Statistical Models

In this section, we will provide all the basic notions of multi-parameter quantum metrology that are needed for our goals. We refer to the following references [12,13,14,15] for more explanations and technical details on this topic.

The aim of local estimation theory is to set the ultimate bounds on how precisely the considered parameters can be estimated. For this purpose, general bounds have been proposed [36,44,45] which depend on the quantum statistical model $\varrho_{𝛌}$ only, that is on the family of quantum states defined in terms of the set of parameters $\lambda =(\lambda_{1},\ldots \lambda_{n})$ to be estimated. In particular one introduces, respectively, the SLD operators $L_{\mu }^{S}$, and the RLD operators $L_{\mu }^{R}$ via the equations

(1) $$\begin{array}{cc} \partial_{\mu }\varrho_{𝛌} & =\frac{L_{\mu }^{S}\;\varrho_{𝛌}+\varrho_{𝛌}\;L_{\mu }^{S}}{2}\; \end{array}$$

(2) $$\begin{array}{cc} \partial_{\mu }\varrho_{𝛌} & =\varrho_{𝛌}\;L_{\mu }^{R}\; \end{array}$$

where $\partial_{\mu }$ corresponds to the partial derivative with respect to the μ-th parameter $\lambda_{\mu }$. It is then possible to derive the following (measurement independent) matrix quantum Cramér-Rao bounds (CRB), bounding the covariance matrix V(λ) of any locally unbiased estimator [39,40,46]

(3) $$V\left(\lambda \right)\geq Q{\left(\lambda \right)}^{-1},\;\;V\left(\lambda \right)\geq J{\left(\lambda \right)}^{-1}$$

where the corresponding SLD Q(λ) and RLD J(λ) quantum Fisher information (QFI) matrices elements are

(4) $$\begin{array}{cc} Q_{\mu \nu }\left(\lambda \right) & =\mathrm{Tr}\left[\varrho_{𝛌}\frac{L_{\mu }^{S}\;L_{\nu }^{S}+L_{\nu }^{S}\;L_{\mu }^{S}}{2}\right]\; \end{array}$$

(5) $$\begin{array}{cc} J_{\mu \nu }\left(\lambda \right) & =\mathrm{Tr}[\varrho_{𝛌}\;L_{\nu }^{R}\;L_{\mu }^{R†}]\; \end{array}$$

In the single-parameter case, the SLD-bound is tight and it is always possible to saturate it by performing the optimal measurement that in turn corresponds to projecting on the eigenstates of the SLD operator $L_{\lambda }^{S}$.

In the multi-parameter scenario, it is common to rewrite the matrix bounds into scalar CRBs by introducing a positive, real *weight matrix* W, leading to the inequalities

(6) $$\begin{array}{c} \mathrm{Tr}\left[W\;V\right]\geq C^{S}\left(\lambda ,W\right),\;\mathrm{Tr}\left[W\;V\right]\geq C^{R}\left(\lambda ,W\right)\; \end{array}$$

where the scalar SLD- and RLD-CRBs respectively read

(7) $$\begin{array}{cc} C^{S}\left(\lambda ,W\right) & =\mathrm{Tr}[WQ^{-1}]\; \end{array}$$

(8) CR(λ,W)=Tr[WRe(J−1)]+||WIm(J−1)W||1

with ${\left||A|\right|}_{1}=\mathrm{Tr}\left[\sqrt{A^{†}A}\right]$ denoting the trace norm of the matrix A.

Holevo derived a tighter scalar bound $C^{H}\left(𝛌,W\right)$ in [38,39] via the following minimization:

(9) $$\begin{array}{cc} C^{H}\left(\lambda ,W\right) & =\min_{U\in S^{d},X\in X_{𝛌}}\left[\mathrm{Tr}[W\;U]\;\;|\;\;U\geq Z[X]\right] \end{array}$$

(10) =minX∈X𝛌Tr[WReZ[X]]+∥WImZ[X]W∥1

where $S^{d}$ denotes the set of real symmetric *d*-dimensional matrices, and the Hermitian d×d matrix Z is defined via its elements

(11) $$\begin{array}{c} Z_{\mu \nu }\left[X\right]=\mathrm{Tr}\left[\varrho_{𝛌}X_{\mu }X_{\nu }\right]\; \end{array}$$

with the collection of operators X belonging to the set

(12) $$\begin{array}{c} X_{𝛌}=\left\{X=\left(X_{1},\cdots ,X_{d}\right)\;\;|\;\;\mathrm{Tr}\left[\left(\partial_{\mu }\varrho_{𝛌}\right)X_{\nu }\right]=\delta_{\mu \nu }\right\}\; \end{array}$$

In fact, the following chain of inequalities holds:

(13) $$\begin{array}{c} \mathrm{Tr}\left[W\;V\right]\geq C^{H}\left(\lambda ,W\right)\geq \max\left[C^{S}\left(\lambda ,W\right),C^{R}\left(𝛌,W\right)\right]\; \end{array}$$

The Holevo bound is achievable if one considers the corresponding asymptotic model that is by optimizing over collective measurements on an asymptotically large number of copies of the state, $\varrho_{𝛌}^{⊗n}={⨂}_{j=1}^{n}\varrho_{𝛌}$, with n→∞ [40,47,48,49]. It has been proved that there are instances where the Holevo bound is also achieved in the single-copy scenario: this is the case for example of pure state models [50] and displacement estimation with Gaussian states [39].

The SLD- and the Holevo-bound stand out as the most exploited tools in order to characterize a multi-parameter estimation problem. On the one hand, $C^{S}\left(\lambda ,W\right)$ is the straightforward generalization of the single-parameter bound, and it is typically easy to calculate. On the other hand, $C^{H}\left(\lambda ,W\right)$, despite it being difficult to calculate because of the complicated minimization procedure, is always more informative than $C^{S}\left(\lambda ,W\right)$, and it is achievable, at least for the asymptotic model. More recently it has been shown that the Holevo bound can be also upper bounded in terms of the SLD-bound, as follows [42,51,52]:

(14) $$\begin{array}{cc} C^{S}\left(\lambda ,W\right) & \leq C^{H}\left(\lambda ,W\right) \end{array}$$

(15) $$\begin{array}{cc} \; & \leq C^{S}\left(\lambda ,W\right)+{∥\sqrt{W}\;Q^{-1}\;D\;Q^{-1}\;\sqrt{W}∥}_{1} \end{array}$$

(16) $$\begin{array}{cc} \; & \leq \left(1+R\right)\;C^{S}\left(\lambda ,W\right)\leq 2\;C^{S}\left(\lambda ,W\right)\;. \end{array}$$

In the chain of inequalities above, we have introduced two new objects: the (asymptotic) *incompatibility* matrix D, also known as mean Uhlmann curvature [53], with elements

(17) $$\begin{array}{c} D_{\mu \nu }=-\frac{i}{2}\mathrm{Tr}\left[\varrho_{𝛌}\left[L_{\mu }^{S},L_{\nu }^{S}\right]\right]\; \end{array}$$

and the *quantumness* measure

(18) $$\begin{array}{c} R=\left||i\;Q^{-1}D\right|{|}_{\infty }\; \end{array}$$

where ${\left|\left|A\right|\right|}_{\infty }$ denotes the largest eigenvalue of the matrix A.

### 2.1. On the Quantumness Parameter R

The figure of merit R introduced in [42] via the eq. (18) is the main focus of our work. As we will describe below by revising and deriving some of its fundamental properties, R quantifies the *quantumness* of a multi-parameter quantum statistical model, or more in detail, the (asymptotical) incompatibility of the parameters to be estimated.

As it is clear from its definition, R is well defined only for quantum statistical models $\varrho_{𝛌}$ having a non-singular SLD-QFI matrix, and we will thus avoid these pathological cases. Here below we present a list of its properties (notice that we will provide the proof only for property [P5], as it was not presented in [42]):

**[P1]**  the quantumness measure R is bounded as follows
  (19) $$\begin{array}{c} 0\leq R\leq 1\;. \end{array}$$
**[P2]**  one has that
  (20) $$\begin{array}{c} R=0\;\;\Leftrightarrow D=0\;, \end{array}$$
  that is if and only if the *weak compatibility* condition for the SLD operators holds,
  $$\mathrm{Tr}[\varrho_{𝛌}\left[L_{\mu }^{S},L_{\nu }^{S}\right]]=0\;.$$
  Consequently, in this case, one has that $C^{H}\left(\lambda ,W\right)=C^{S}\left(\lambda ,W\right)$ for all weight matrices W, and thus the quantum statistical model is said to be *asympotically classical*: the SLD-bound is asymptotically achievable via collective measurements on $\varrho_{𝛌}^{⊗n}={⨂}_{j=1}^{n}\varrho_{𝛌}$, with n→∞ [54].
**[P3]**  Given any possible weight matrix W, the following inequality holds:
  (21) $$\begin{array}{c} \Delta C(\lambda ,W)\leq R\;, \end{array}$$
  that is the quantumness R is an upper bound for the renormalized difference between Holevo and SLD-bound
  (22) $$\begin{array}{c} \Delta C\left(\lambda ,W\right)=\frac{C^{H}\left(\lambda ,W\right)-C^{S}\left(\lambda ,W\right)}{C^{S}\left(\lambda ,W\right)}\;. \end{array}$$
**[P4]**  If the number of parameters to be estimated is n=2, one has that
  (23) $$\begin{array}{c} R=\sqrt{\frac{\det D}{\det Q}}\;. \end{array}$$
**[P5]**  The quantumness R is invariant under reparametrization of the quantum statistical model: given a new statistical model $\varrho_{\bar{𝛌}}$, such that the new set of *n* parameters are obtained as a function of the original ones, $\bar{\lambda }=f\left(\lambda \right)$, then
  (24) $$\begin{array}{c} R\left(\bar{\lambda }\right)=R\left(\lambda \right)\;. \end{array}$$

**Proof.**  The SLD- and Uhlmann curvatures matrices for the two quantum statistical models are related via the equations

(25) $$\begin{array}{c} Q\left(\bar{\lambda }\right)=B\;Q\left(\lambda \right)\;B^{T},\;\;\;\;\;D\left(\bar{\lambda }\right)=B\;D\left(\lambda \right)\;B^{T}\; \end{array}$$

where the reparametrization matrix B is defined via its elements $B_{\mu \nu }=\partial \lambda_{\nu }/\partial {\bar{\lambda }}_{\mu }$. As a consequence, one can write the corresponding quantumness parameter as

(26) $$\begin{array}{cc} R(\bar{\lambda }) & ={\left|\left|iQ{\left(\bar{\lambda }\right)}^{-1}D\left(\bar{\lambda }\right)\right|\right|}_{\infty } \end{array}$$

(27) $$\begin{array}{cc} \; & ={\left|\left|i{\left(B^{T}\right)}^{-1}Q{\left(\lambda \right)}^{-1}B^{-1}BD\left(\lambda \right)B^{T}\right|\right|}_{\infty } \end{array}$$

(28) $$\begin{array}{cc} \; & ={\left|\left|i{\left(B^{T}\right)}^{-1}Q{\left(\lambda \right)}^{-1}D\left(\lambda \right)B^{T}\right|\right|}_{\infty } \end{array}$$

(29) $$\begin{array}{cc} \; & ={\left|\left|iQ{\left(\lambda \right)}^{-1}D\left(\lambda \right)\right|\right|}_{\infty } \end{array}$$

(30) $$\begin{array}{cc} \; & =R(\lambda )\; \end{array}$$

where the next-to-last equality is satisfied as *similar matrices* (a matrix *A* is similar to a matrix *B* if an invertible matrix *C* exists such that $B=C^{-1}AC$) have the same eigenvalues. One should notice that this result is in fact consistent with the observation that the quantity R does not depend on the weight matrix W: to apply a different weight matrix is formally equivalent to defining a new quantum statistical model via a reparametrization of the set of parameters λ. □

### 2.2. On the Evaluation of the Holevo Bound for Single Qubit Statistical Models

In the next section, we will evaluate the quantumness parameter R and the renormalized difference between Holevo- and SLD-bound ΔC(λ,W) for several multiparameter quantum statistical models for single qubits. In order to calculate ΔC(λ,W), it will be necessary to evaluate both the SLD-bound and the Holevo bound. While the evaluation of the SLD-bound is typically straightforward, as we mentioned before, the minimization needed for the evaluation of the Holevo bound is in general complicated. There are, however, few instances where the Holevo bound can always be easily calculated:

- *Asymptotically classical models*: as previously discussed if D(λ)=0, then one has straightforwardly that $C^{H}\left(\lambda ,W\right)=C^{S}\left(\lambda ,W\right)$.
- *D-invariant models*: if a model is D-invariant (we refer to these references [12,55] for a precise definition and characterization of quantum statistical models, as it goes beyond the scope of this work), then
  (31) $$\begin{array}{cc} C^{H}\left(\lambda ,W\right) & =C^{R}\left(\lambda ,W\right) \end{array}$$
  (32) $$\begin{array}{cc} \; & =C^{S}\left(\lambda ,W\right)+{∥\sqrt{W}\;Q^{-1}\;D\;Q^{-1}\;\sqrt{W}∥}_{1}\;, \end{array}$$
  that is, the Holevo-bound is equal to the RLD-bound and both can be expressed in terms of the SLD matrices Q and D only [43]. It is important to remark that all quantum statistical models corresponding to full state tomography of finite-dimensional quantum system are D-invariant.

If the quantum statistical model does not fall into these classes, the evaluation of $C^{H}\left(\lambda ,W\right)$ may be unfeasible. Several efforts have been made in the literature in order to obtain numerical or even analytical results, at least for some specific classes of quantum states [26,28,56,57,58,59,60,61]. In particular, a closed formula has been derived for all single-qubit two-parameter quantum statistical models [43]. In order to obtain it, we have first to introduce a new quantity, namely

(33) $$\begin{array}{c} C^{Z}\left(\lambda ,W\right)=C^{S}\left(\lambda ,W\right)+{∥\sqrt{W}\;Q^{-1}\;D\;Q^{-1}\;\sqrt{W}∥}_{1}\;. \end{array}$$

The Holevo bound for any two-parameter qubit model under the regularity condition can then be written as

(34) $$C^{H}\left(\lambda ,W\right)=\left\{\begin{array}{cc} C^{R}\left(\lambda ,W\right) & \mathrm{if}\;\;C^{R}\left(\lambda ,W\right)\geq \frac{C^{Z}\left(\lambda ,W\right)+C^{S}\left(\lambda ,W\right)}{2}\\ C^{R}\left(\lambda ,W\right)+S\left(\lambda ,W\right) & \mathrm{otherwise} \end{array}\right.$$

where the function S(λ,W) is non-negative and defined by

(35) $$S\left(\lambda ,W\right):=\frac{{\left[\frac{1}{2}\left(C^{Z}\left(\lambda ,W\right)+C^{S}\left(\lambda ,W\right)\right)-C^{R}\left(\lambda ,W\right)\right]}^{2}}{C^{Z}\left(\lambda ,W\right)-C^{R}\left(\lambda ,W\right)}.$$

From the formulas above, it is apparent that the Holevo bound for two-parameter estimation problems can be written in terms of SLD and RLD operators only.

## 3. *Quantumness* of Single-Qubit Multiparameter Quantum Statistical Models

In this section, we will present the main results of our manuscript. We will consider different quantum statistical models for single qubits, we will evaluate the corresponding quantumness parameter R, and we will compare it with the renormalized difference between Holevo and SLD-bound ΔC(λ,W). We will look in particular for its maximum value, obtained by varying the weight matrix W, i.e.,

(36) $$\begin{array}{c} \Delta C_{\max}=\max_{W>0}\Delta C\left(\lambda ,W\right)\;. \end{array}$$

It is important to remark that this quantity is also invariant under reparametrization, as considering a different weight matrix simply corresponds to considering a new set of parameters to be estimated.

We will start by considering the full-tomography case for both pure and mixed qubit states, corresponding respectively to n=2 and n=3 parameter estimation problems. Then, we will approach several different two-parameter models describing the evolution of qubits into noisy channels, such as phase-diffusion and amplitude damping.

### 3.1. Pure State Model

Given a generic two-parameter pure state model $|\psi_{𝛌}\rangle$, with $\lambda =(\lambda_{1},\lambda_{2})$, the SLD-QFI matrix and the Uhlman curvature matrix can be easily evaluated via the following equations:

(37) Q(λ)=4α+a2Re(c)+abRe(c)+abβ+b2

(38) D(λ)=40Im(c)−Im(c)0

where $a\equiv \langle \partial_{{𝛌}_{1}}\psi_{𝛌}|\psi_{𝛌}\rangle \;\;$, $b\equiv \langle \partial_{{𝛌}_{2}}\psi_{𝛌}|\psi_{𝛌}\rangle \;\;$, $c\equiv \langle \partial_{{𝛌}_{1}}\psi_{𝛌}|\partial_{{𝛌}_{2}}\psi_{𝛌}\rangle \;\;$, $\alpha \equiv \langle \partial_{{𝛌}_{1}}\psi_{𝛌}|\partial_{{𝛌}_{1}}\psi_{𝛌}\rangle \;\;$ and $\beta \equiv \langle \partial_{{𝛌}_{2}}\psi_{𝛌}|\partial_{{𝛌}_{2}}\psi_{𝛌}\rangle$. It follows that

(39) R=|Im(c)|(ab+Re(c))2−4(a2+α)(b2+β).

Here, we are interested in a generic pure qubit state

(40) $$\begin{array}{c} |\psi_{𝛌}\rangle =\cos\frac{\theta }{2}|0\rangle +e^{i\phi }\sin\frac{\theta }{2}|1\rangle \;. \end{array}$$

and thus, in studying the estimation properties of the parameters λ=(θ,ϕ), corresponding to the full tomography of the state. By exploiting the Equations (37) and (38), one can easily evaluate the SLD matrices, obtaining

(41) $$\begin{array}{cc} Q(\lambda ) & =\left(\begin{array}{cc} 1 & 0\\ 0 & \sin^{2}\theta \end{array}\right)\; \end{array}$$

(42) $$\begin{array}{cc} D(\lambda ) & =\left(\begin{array}{cc} 0 & -\sin\theta \\ \sin\theta & 0 \end{array}\right)\;. \end{array}$$

Consequently, one obtains $R=\sqrt{\det Q/\det D}=1$, that is, according to this measure, the parameters are maximally incompatible for any possible values of θ and ϕ (notice that we are not considering the case where the SLD-QFI matrix is singular, θ={0,π}).

We are now interested in evaluating the SLD- and the Holevo bound; in particular, we will restrict to diagonal weight matrices that can be generically written as W=diag(1,w), with w>0. It has been demonstrated that any pure state qubit model is D-invariant [43]. While, in general, the RLD operators for a pure state model are not well defined, it was shown in [62] that the formula in Equation (32) is still valid also in the limit of pure states. Consequently, one can evaluate both the SLD- and the Holevo-bound as

(43) $$\begin{array}{cc} C^{S}\left(\lambda ,W\right) & =\mathrm{Tr}\left[WQ^{-1}\right]=1+\frac{w}{\sin^{2}\theta }\; \end{array}$$

(44) $$\begin{array}{cc} C^{H}\left(\lambda ,W\right) & =\mathrm{Tr}\left[WQ^{-1}\right]+{∥\sqrt{W}\;Q^{-1}\;D\;Q^{-1}\;\sqrt{W}∥}_{1}={\left(1+\frac{\sqrt{w}}{\sin\theta }\right)}^{2}\; \end{array}$$

leading to

(45) $$\begin{array}{c} \Delta C\left(\lambda ,W\right)=\frac{2\sqrt{w}\sin\theta }{w+\sin^{2}\theta }\;. \end{array}$$

Remarkably, one observes that, by choosing $w=w^{\left(\max\right)}=\sin^{2}\theta$, one has $\Delta C_{\max}=\Delta C\left(\lambda ,W\right)=1$, that is, for any value of the parameters λ, it is possible to find a diagonal weight matrix such that the renormalized difference between Holevo and SLD-bound is equal to the quantumness R (and, in this case, to its maximum value 1). Remarkably, the optimal weight matrix is equal to the SLD-QFI matrix, W=Q(λ) that is when the parameters are weighted according to the Bures metric defined by the statistical model [63]. We have thus evidence that, for this model, the bound ΔC(λ,W)≤R is in fact tight, and thus the quantity R is a good measure of incompatibility of the two parameters θ and ϕ. On the other hand, we also observe how, at fixed weight matrix W, the difference between Holevo- and SLD-bound may be much smaller than the one predicted by the quantumness R.

We finally remark that any other two-parameter estimation problem involving pure qubit states will be characterized by the same figures of merit R and $\Delta C_{\max}$. We indeed proved via the property **[P5]** that the quantumness parameter R is invariant under reparametrization. Consequently, the result above also holds for the (unitary) quantum statistical model corresponding to pure states

(46) $$\begin{array}{c} |\psi_{\bar{\lambda }}\rangle =e^{i\lambda_{x}\sigma_{1}+i\lambda_{z}\sigma_{3}}|\psi_{0}\rangle \;,\;\;\;\bar{\lambda }=\left(\lambda_{x},\lambda_{z}\right)\;, \end{array}$$

which is corresponding to the estimation of the two *phases* $\lambda_{x}$ and $\lambda_{z}$ due to the unitary evolution $U=e^{i\lambda_{x}\sigma_{1}+i\lambda_{z}\sigma_{3}}$ applied on a generic initial state $|\psi_{0}\rangle$.

### 3.2. Full Tomography of a Qubit Mixed State

We now consider the quantum statistical model corresponding to a generic mixed qubit state

(47) $$\begin{array}{c} \varrho_{𝛌}=\frac{1}{2}\left(1+\sum_{j=1}^{3}\gamma_{j}\sigma_{j}\right)\; \end{array}$$

where the matrices $\sigma_{j}$ denote Pauli matrices and

(48) $$\begin{array}{c} \gamma_{1}=r\sin\theta \cos\phi \;,\;\;\;\;\;\gamma_{2}=r\sin\theta \sin\phi \;,\;\;\;\;\;\gamma_{3}=r\cos\theta \; \end{array}$$

and we thus consider the set of parameters λ=(r,θ,ϕ) characterizing the vector in the Bloch sphere corresponding to the state $\varrho_{𝛌}$. The SLD operators can be easily evaluated by solving the corresponding Lyapunov equations, yielding the matrices

(49) $$\begin{array}{cc} Q(\lambda ) & =\left(\begin{array}{ccc} 1/(1-r^{2}) & 0 & 0\\ 0 & r^{2} & 0\\ 0 & \; & r^{2}\sin\theta^{2} \end{array}\right)\; \end{array}$$

(50) $$\begin{array}{cc} D(\lambda ) & =\left(\begin{array}{ccc} 0 & 0 & 0\\ 0 & 0 & r^{3}\sin\theta \\ 0 & -r^{3}\sin\theta & 0 \end{array}\right)\; \end{array}$$

We will investigate the regime where the SLD-QFI matrix is not singular, that is, avoiding the cases of maximally mixed (r=0) or maximally pure (r=1) states, and for values of the azimuthal angle θ={0,π}. By evaluating the quantumness parameter via its definition (18), one obtains R=r, that is, the quantumness is in fact equal to the length of the Bloch vector characterizing the qubit.

As in the previous case, we now focus on diagonal weight matrices $W=\mathrm{diag}(1,w_{\theta },w_{\phi })$ that can be parametrized in terms of two positive real numbers $w_{\theta }$ and $w_{\phi }$. In addition, in this case, one proves that the model is D-invariant and, as a consequence, one can evaluate both the SLD- and the Holevo bound

(51) $$\begin{array}{cc} C^{S}\left(𝛌,W\right) & =\frac{\left(r^{2}-r^{4}+w_{\theta }\right)\sin^{2}\theta +w_{\phi }}{r^{2}\sin^{2}\theta }\; \end{array}$$

(52) $$\begin{array}{cc} C^{H}\left(𝛌,W\right) & =\frac{\left(r^{2}-r^{4}+w_{\theta }\right)\sin^{2}\theta +2r\sqrt{w_{\theta }w_{\phi }}\sin\theta +w_{\phi }}{r^{2}\sin^{2}\theta }\; \end{array}$$

corresponding to

(53) $$\begin{array}{c} \Delta C\left(𝛌,W\right)=\frac{2r\sqrt{w_{\theta }w_{\phi }}\sin\theta }{\left(r^{2}-r^{4}+w_{\theta }\right)\sin^{2}\theta +w_{\phi }}\;. \end{array}$$

One can then check that it is not possible to find a couple of real positive weights $\left(w_{\theta },w_{\phi }\right)$ such that ΔC(λ,W)=r, unless we have r=1, that is, for pure states, as already shown in the previous section. In particular, we also notice that, in this case, by choosing as the weight matrix the SLD-QFI matrix, W=Q(λ); one obtains ΔC(λ,W)=2r/3=2R/3, and thus weighting the parameters according to the Bures metric is not the optimal choice. However, we can check that, for generic *r*, by setting $w_{\theta }$ equal to the real part of the solution of the equation ΔC(λ,W)=r, that is,

(54) $$\begin{array}{c} w_{\theta }^{\left(\max\right)}=\frac{w_{\phi }+\left(r^{4}-r^{2}\right)\sin^{2}\theta }{\sin^{2}\theta }\; \end{array}$$

one obtains

(55) $$\begin{array}{c} \Delta C\left(\lambda ,W\right)=\frac{r\sqrt{w_{\phi }^{2}+w_{\phi }\left(r^{4}-r^{2}\right)\sin^{2}\theta }}{w_{\phi }}\;, \end{array}$$

that, in the limit of $w_{\phi }\to \infty$, gives ΔC(λ,W)→r. However, as also suggested by some numerics, it is not necessary to consider an *infinite weight* $w_{\phi }$, as it is easy to find diagonal weight matrices W such that the corresponding renormalized difference ΔC(λ,W) is ϵ-close to the quantumness parameter R=r. In general, we can conclude that, also in this case, the bound for ΔC(λ,W) is *almost* tight and thus the parameter R is in fact a good measure of quantumness for the statistical model.

### 3.3. Simultaneous Estimation of Frequency and Dephasing Rate

We now consider the evolution of a qubit system corresponding to a simultaneous rotation around the *z*-axis with frequency ω and dephasing with rate γ, ruled by the following Lindblad master equation:

(56) $$\dot{\varrho }=-i\frac{\omega }{2}\left[\sigma_{3},\varrho \right]+\frac{\gamma }{2}D\left[\sigma_{3}\right]\varrho \;$$

where we have defined the superoperator

(57) $$D\left[A\right]\varrho =A\varrho A^{†}-\frac{1}{2}\left(A^{†}A\varrho +\varrho A^{†}A\right)\;.$$

Given a generic initial pure state $|\psi_{0}\rangle =\cos\left(\theta /2\right)|0\rangle +e^{i\phi }\sin\left(\theta /2\right)|1\rangle$, the evolved density matrix can be analytically evaluated, and we are going to consider it as our quantum statistical model:

(58) $$\varrho_{𝛌}=\left(\begin{array}{cc} \cos^{2}\left(\theta /2\right) & \frac{1}{2}e^{-(\gamma -i\omega )t-i\phi }\sin\theta \\ \frac{1}{2}e^{-(\gamma +i\omega )t+i\phi }\sin\theta & \sin^{2}\left(\theta /2\right) \end{array}\right),\;\;\;\;\;𝛌=\left(\omega ,\gamma \right)\;.$$

As it clear from the formula above, the evolution time *t* is just a multiplicative factor for both the parameters we want to estimate, that is, the frequency ω and the dephasing rate γ, and thus will not play any fundamental role in the evaluation of our figures of merit. The SLD- and the RLD-operators can be evaluated without difficulties by solving the corresponding defining Equations (1) and (2). The corresponding QFI matrices and the Ulhman curvature matrix then reads

(59) $$\begin{array}{cc} Q(\lambda ) & =\left(\begin{array}{cc} \frac{4t^{2}\sin^{2}\theta }{e^{2\gamma t}-1}\; & 0\\ 0 & 4e^{-2\gamma t}t^{2}\sin^{2}\theta \end{array}\right)\; \end{array}$$

(60) $$\begin{array}{cc} D(\lambda ) & =\left(\begin{array}{cc} 0 & 4e^{-2\gamma t}t^{2}\;\cos\theta \sin^{2}\theta \\ -4e^{-2\gamma t}t^{2}\;\cos\theta \sin^{2}\theta \; & 0 \end{array}\right)\; \end{array}$$

(61) $$\begin{array}{cc} J(\lambda ) & =\left(\begin{array}{cc} \frac{4t^{2}}{e^{2\gamma t}-1} & \frac{4it^{2}\cos\theta }{e^{2\gamma t}-1}\\ -\frac{4it^{2}\cos\theta }{e^{2\gamma t}-1} & \frac{4t^{2}}{e^{2\gamma t}-1} \end{array}\right)\;. \end{array}$$

By exploiting Equation (23), we obtain quantumness parameter R

(62) $$R=\left|\cos\theta \right|\sqrt{1-e^{-2\gamma t}}\;$$

showing how the maximum incompatibility at fixed γ is obtained in the limit of θ→0 and θ→π, that is, when the model is not well defined, as the SLD-QFI is singular. In this limit, in fact, the initial state is an eigenstate of $\sigma_{3}$ and thus the state remains unchanged during the evolution due to the master Equation (56) without acquiring any information on the parameters. As regards the behavior of R as a function of the dephasing parameter γ, it is easy to check that R monotonically increases with γ, and thus by decreasing the purity of the corresponding quantum statistical model $\varrho_{𝛌}$; this observed behavior is opposite to what we have discussed previously for state tomography, where we in fact found that the quantumness R coincided with the purity of the qubit state $\varrho_{𝛌}$.

**Figure 1:**
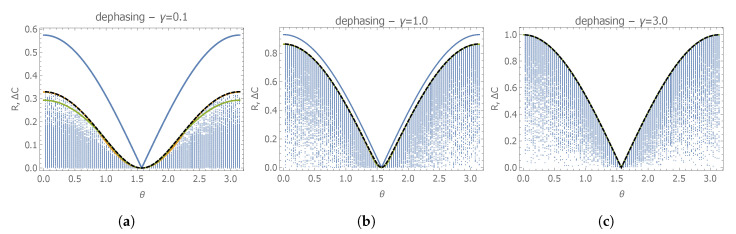
Plots of the quantumness parameter R (blue solid line) and of the renormalized difference ΔC(λ,W) for simultaneous estimation of frequency and dephasing rate, as a function of the initial state parameter θ. The black dashed line corresponds to ΔC(λ,W) with an optimized diagonal weight matrix **W** for every value of θ. Yellow and green correspond to ΔC(λ,W) with diagonal weight matrix optimized, respectively, for θ={π/3,2π/3} (in some of the plots, these curves are not visible as they are perfectly superimposed by the dashed-black line corresponding to the optimized ΔC). Blue points correspondl to ΔC(λ,W) evaluated for random generic weight matrices. The three plots correspond to different values of the dephasing rate: (**a**) γt=0.1; (**b**) γt=1.0; (**c**) γt=3.0.

For this quantum statistical model, the Holevo bound has to be evaluated via Equation (34), as the model is neither asymptotically classical nor D-invariant. In particular, as in the previous examples, we started by considering diagonal weight matrices W=diag(1,w). The optimal $w=w^{\left(\max\right)}$ maximizing ΔC(λ,W) depends on the initial state of the qubit $|\psi_{0}\rangle$, and in particular on its angle θ. Remarkably, we have strong numerical evidence that, in the limit θ→0 or θ→π, we have $w^{\max}=\Delta C{\left(\lambda ,W\right)}^{2}$, which is exactly equal to the square of the corresponding renormalized difference between Holevo and SLD-bound. We have also generated random generic (non-diagonal) weight matrix and, as one can see in Figure 1, we obtain that optimizing over diagonal W is enough to obtain $\Delta C_{\max}$; that is, in general, one obtains values of ΔC smaller than the one optimized on diagonal-weight matrices (we also notice that W=Q(𝛌) is not optimal at fixed γ). Moreover, we also observe that $\Delta C_{\max}$ and R share the same qualitative behavior as a function of the parameters θ and γ. However, we also observe that, for small values of the dephasing rate γ, ΔC is strictly smaller than R; that is, the quantumness parameter seems to overestimate the incompatibility of frequency and dephasing. Only by increasing γ can we find that the gap between these two quantities goes to zero for all values of θ (in particular, we find that this is already the case for γ=1.5). In the limit of γ→∞, the optimal weight matrix becomes the identity W=1, a choice that is equivalent to weighting the parameters according to the Bures metric (in this limit, the ratio between the two diagonal non-zero elements of the SLD-QFI matrix ${\left[Q\left(𝛌\right)\right]}_{2,2}/{\left[Q\left(𝛌\right)\right]}_{1,1}$ goes indeed to one).

### 3.4. Simultaneous Estimation of Frequency and Amplitude Damping Rate

In this last example, we consider a different noisy evolution, ruled by the Lindblad master equation:

(63) $$\dot{\varrho }=-i\frac{\omega }{2}\left[\sigma_{3},\varrho \right]+\gamma D\left[\sigma_{-}\right]\varrho \;,\;\;\;\;\;\;\mathrm{where}\;\;\sigma_{-}=\frac{\sigma_{1}-i\sigma_{2}}{2}\;$$

corresponding to simultaneous rotation around the *z*-axis with frequency ω and amplitude damping with rate γ. In addition, in this case, the equation can be analytically solved, and, for a generic initial state $|\psi_{0}\rangle =\cos\left(\theta /2\right)|0\rangle +e^{i\phi }\sin\left(\theta /2\right)|1\rangle$, we obtain our quantum statistical model

(64) $$\varrho_{𝛌}=\left(\begin{array}{cc} 1-e^{\gamma t}\sin^{2}\left(\theta /2\right) & \frac{1}{2}e^{-(\gamma /2+i\omega )t-i\phi }\sin\theta \\ \frac{1}{2}e^{-(\gamma /2-i\omega )t+i\phi }\sin\theta & e^{\gamma t}\sin^{2}\left(\theta /2\right) \end{array}\right),\;\;\;\;\;𝛌=\left(\omega ,\gamma \right)\;.$$

As in the previous example, the evolution time *t* is just a multiplicative factor for both parameters and thus is not going to play any role in our results.

The equations for the SLD- and the RLD-operators can be easily solved, and, consequently, we have been able to evaluate analytically the following matrices:

(65) $$\begin{array}{cc} Q(\lambda ) & =\left(\begin{array}{cc} \frac{e^{-\gamma t}t^{2}\sin^{2}\left(\frac{\theta }{2}\right)\left(1+\cos\theta -2e^{\gamma t}\right)}{2(1-e^{\gamma t})}\; & 0\\ 0 & 4e^{-\gamma t}t^{2}\sin^{2}\theta \end{array}\right)\; \end{array}$$

(66) $$\begin{array}{cc} D(\lambda ) & =\left(\begin{array}{cc} 0 & e^{-2\gamma t}t^{2}\;\sin^{2}\theta \left(\cos\theta -1-e^{\gamma t}\right)\\ -e^{-2\gamma t}t^{2}\;\sin^{2}\theta \left(\cos\theta -1-e^{\gamma t}\right)\; & 0 \end{array}\right)\; \end{array}$$

(67) $$\begin{array}{cc} J(\lambda ) & =\left(\begin{array}{cc} \frac{t^{2}\left(e^{\gamma t}+4+\left(e^{\gamma t}-4\right)\cos\theta \right)}{8\sin^{2}\left(\frac{\theta }{2}\right)\left(e^{\gamma t}-1\right)} & \frac{it^{2}\cos^{2}\left(\frac{\theta }{2}\right)\left(\cos\theta -1-e^{\gamma t}\right)}{\left(e^{\gamma t}-1\right)\sin^{2}\left(\frac{\theta }{2}\right)}\\ -\frac{it^{2}\cos^{2}\left(\frac{\theta }{2}\right)\left(\cos\theta -1-e^{\gamma t}\right)}{\left(e^{\gamma t}-1\right)\sin^{2}\left(\frac{\theta }{2}\right)}\; & \frac{4e^{\gamma t}t^{2}\cos^{2}\left(\frac{\theta }{2}\right)}{e^{\gamma t}-1} \end{array}\right)\;. \end{array}$$

The quantumness parameter R can be straightforwardly evaluated by exploiting Equation (23), obtaining

(68) $$R=e^{-\gamma t}\cos\frac{\theta }{2}\left(1+e^{\gamma t}-\cos2\theta \right)\sqrt{\frac{2(1-e^{\gamma t})}{1-2e^{\gamma t}+\cos\theta }}\;.$$

As it is clear from the equations, all these quantities, and, in particular, the parameter R depends only on the initial angle θ and on the amplitude damping γt. In particular, at fixed γt, one can show that R has a monotonous decreasing behavior with θ, from its maximum value R=1 for θ→0, to its minimum value R=0 for θ→π. It is important to notice that these extremal cases correspond to the values of θ that make the SLD-QFI singular (for θ→0, all the elements of the SLD-QFI matrix actually become identical to zero). As regards the behavior as a function of γ, we observe that R is monotonically increasing for γt∈[0,ln2], and then monotonically decreasing in the interval γt∈(ln2,∞). As in the previous example, we find that this behavior is opposite to the behavior of the purity of the quantum state $\varrho_{𝛌}$, which is indeed decreasing for γt∈[0,ln2] and then increasing for larger values of γ.

**Figure 2:**
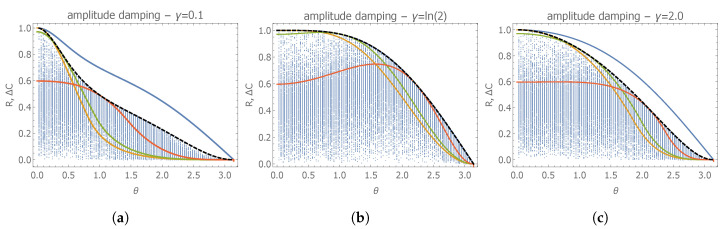
Plots of the quantumness parameter R (blue solid line) and of the renormalized difference ΔC(λ,W) for simultaneous estimation of frequency and amplitude damping rage, as a function of the initial state parameter θ. The black dashed line corresponds to ΔC(λ,W) with an optimized diagonal weight matrix **W** for every value of θ. Yellow, green, and red lines correspond to ΔC(λ,W) with diagonal weight matrix optimized, respectively, for θ={π/3,2π/3}. Blue points correspond to ΔC(λ,W) evaluated for random generic weight matrices. The three plots correspond to different values of the amplitude damping rate: (**a**) γt=0.1; (**b**) γt=ln2; (**c**) γt=2.0.

In addition, this quantum statistical model is neither asymptotically classical nor D-invariant. Consequently, the Holevo-bound and the renormalized difference ΔC(λ,W) have been evaluated numerically by exploiting Equation (34). We started again our investigation by considering a diagonal weight matrix W=diag(1,w), and we have found that, also by optimizing over the free parameter *w* for fixed initial state $|\psi_{0}\rangle$, the renormalized difference ΔC(λ,W) is in general smaller than the quantumness R. As before, in order to assess the generality of this result, we have generated numerically thousands of non-diagonal random weight matrices. As one can observe from Figure 2, we have observed that, in general, the maximum value of ΔC obtained via diagonal weight matrices is in fact an upper bound for generic weight matrices. We thus assume that, by optimizing over diagonal matrices, one obtains $\Delta C_{\max}$. Observing the figure, we have evidence that, also in this case, the qualitative behavior of $\Delta C_{\max}$ and R as a function of the different parameters is the same; however, it is possible to observe a non-zero gap between R and ΔC, and thus the parameter R in general overestimates the degree of incompatibility of the two parameters. In particular, we observe that the gap is always closed in the limit of θ→0, that is, when R=1, and the optimal weight matrix parameter takes the value w≈16. We observe that the gap is also closed for all values of θ by taking γt=ln2, where we obtain

(69) $$\begin{array}{c} R=\Delta C_{\max}=\cos\left(\theta /2\right)\sqrt{\frac{3-\cos\theta }{2}}\;. \end{array}$$

In this case, the optimal (diagonal) weight parameter reads

(70) $$\begin{array}{c} w^{\left(\max\right)}=\frac{{\left[Q\left(𝛌\right)\right]}_{2,2}}{{\left[Q\left(𝛌\right)\right]}_{1,1}}=\frac{16(1+\cos\theta )}{3-\cos\theta }\; \end{array}$$

that is, the optimality is obtained by weighting the parameters according to the corresponding Bures metric. We have thus found another instance where the bound is tight, and the optimal weight matrix is equal (or equivalently proportional) to the SLD-QFI matrix Q(λ) (we also remark that, for values of γt≠ln2, that is, whenever the bound is not tight, the optimal weight matrix differs from Q(𝛌)).

### 3.5. Asymptotically Classical Models

Here, we will list a couple of examples of Lindblad master equations for qubits involving two parameters, whose solutions correspond to quantum statistical models $\varrho_{𝛌}$ that are asymptotically classical, that is, whose quantumness parameter R is equal to zero.

- *Simultaneous estimation of frequency and depolarizing channel rate*, corresponding to the master equation
  (71) $$\begin{array}{c} \dot{\varrho }=-i\frac{\omega }{2}\left[\sigma_{3},\varrho \right]+\frac{\gamma }{2}\left(\frac{1}{3}\sum_{i=1}^{3}\sigma_{i}\;\varrho \;\sigma_{i}-\varrho \right),\;\;\;\;\;\lambda =\left(\omega ,\gamma \right) \end{array}$$
- *Simultanoues estimation of amplitude damping and dephasing rates*, corresponding to the master equation
  (72) $$\begin{array}{c} \dot{\varrho }=\gamma_{\mathrm{ad}}D\left[\sigma_{-}\right]\varrho +\frac{\gamma_{\mathrm{deph}}}{2}D\left[\sigma_{3}\right]\varrho ,\;\;\;\;\;\lambda =\left(\gamma_{\mathrm{ad}},\gamma_{\mathrm{deph}}\right) \end{array}$$

## 4. Discussion and Conclusions

In this paper, we have studied in detail the quantumness of multiparameter quantum statistical models for qubit systems, defined as the incompatibility of the parameters to be jointly estimated. In particular, we have evaluated the renormalized difference $\Delta C_{\max}$ between the Holevo- and the SLD-bound optimized over all the possible weight matrices, and its upper bound given by the quantumness measure R. Our results confirm that R is a useful and practical tool to characterize the properties of the quantum statistical model: (i) we have shown some examples where in fact $\Delta C_{\max}=R$, and remarkably we have found that in these cases the weight matrix maximizing ΔC always corresponds to the Bures metric induced by the quantum statistical model; (ii) we have observed that in general the two quantities, R and $\Delta C_{\max}$, have the same qualitative behavior. In particular they show a peculiar counterintuitive dependence on the purity of the quantum states $\varrho_{𝛌}$: in the quantum state tomography scenario, both R and $\Delta C_{\max}$ are monotonically increasing with the purity, while in the two noisy models induced by the Markovian master equation, we observe the opposite behavior, and larger values of R (or $\Delta C_{\max}$) are obtained for low purity states.

However our results give also clear evidence that the bound is in general not always tight, i.e., it is possible to find several examples where R overestimates the actual degree of incompatibility of the parameters, and thus the evaluation of the Holevo-bound is needed to properly assess and quantify this property of the quantum statistical model.

We believe that our work together with other complementary approaches, such as the one pursued in [64] where trade-off surfaces are derived via the SLD- and the Holevo-bound, will help in shedding new light on the relationship between quantum uncertainty relations, incompatibility and multi-parameter quantum metrology.
